# The Treatment of Vasomotor Rhinitis With Intranasal Corticosteroids

**DOI:** 10.1097/WOX.0b013e3181af7c93

**Published:** 2009-08-15

**Authors:** Eli O Meltzer

**Affiliations:** 1 Allergy & Asthma Medical Group & Research Center; Clinical Professor of Pediatrics, University of California, San Diego; Allergy & Asthma Medical Group & Research Center, 9610 Granite Ridge Dr., Suite B San Diego, CA

**Keywords:** nonallergic rhinitis, vasomotor rhinitis, intranasal corticosteroids, beclomethasone dipropionate, budesonide, fluticasone propionate, mometasone furoate

## Abstract

**Objective:**

Intranasal steroids (INS) are firmly established as the therapy for choice for allergic rhinitis, but their role in vasomotor rhinitis (VMR) is not fully characterized. This review examines the potential mechanisms of action and reported efficacy of INS in patients with VMR.

**Results:**

INS, through intracellular activation of the glucocorticoid receptor, down-regulate the recruitment and activation of inflammatory cells (T-lymphocytes, eosinophils, mast cells, basophils, neutrophils, macrophages), increase degradation of neuropeptides, and reduce epithelial cell activity, vascular permeability, and chemokine secretion. It is likely that more than vasoconstriction is responsible for the clinical effects of INS.

Eight INS can be prescribed for rhinitis in the US; only 4 have been studied for VMR. Seventy-four percent of patients treated with beclomethasone dipropionate considered themselves symptom-free or greatly improved versus 31% with placebo. Budesonide significantly reduced rhinitis symptoms and methacholine-induced nasal secretions compared with placebo. Fluticasone propionate compared with placebo provided significantly greater relief from nasal obstruction; computed tomographic scans showed significant reductions in the mucosal area of the lower turbinates. Mometasone furoate produced numerically better rhinitis symptom scores and, when discontinued, lower relapse rates than placebo.

**Conclusion:**

Data supports INS as beneficial pharmacotherapy for VMR.

## Introduction

Vasomotor rhinitis (VMR, also referred to as idiopathic rhinitis) is diagnosed in a heterogeneous group of patients with chronic nasal symptoms that are not immunologic or infectious in origin and usually not associated with nasal eosinophilia. Although the term vasomotor implies increased neural efferent traffic to the blood vessels supplying the nasal mucosa, this has never been proven [1]. However, it is suggested that neurogenic reflex mechanisms initiated by environmental factors may be involved. There could be an imbalance of the sympathetic and parasympathetic nervous systems, with parasympathetic hyperactivity and sympathetic hypoactivity resulting in rhinorrhea and nasal congestion. Indirect evidence also postulates that C-fibers may play a role in the pathophysiology of VMR [2]. According to this second theory, in nonallergic, noninfectious perennial rhinitis an overactive nonadrenergic, noncholinergic system causes neurogenic inflammation, resulting in increased neuropeptides [3]. The recently updated rhinitis parameters developed by the Joint Task Force on Practice Parameters, representing the American Academy of Allergy, Asthma and Immunology, the American College of Allergy, Asthma and Immunology, and the Joint Council of Allergy, Asthma and Immunology state, "intranasal corticosteroids are effective in the treatment of vasomotor rhinitis."[1]

## Intranasal corticosteroids

Intranasal corticosteroids (INSs) are effective therapeutic agents. In recent years, increased understanding of corticosteroid and glucocorticoid receptor pharmacology has enabled the development of molecules designed specifically to achieve potent, localized activity with minimal risk of systemic exposure.

Systemic corticosteroids, which were developed in the 1950s, are effective in treating various rhinopathies; but the high risk of serious toxicity with long-term administration has hindered their usefulness [4]. Initial attempts to deliver compounds such as hydrocortisone and dexamethasone directly into the airways were only partially successful [5]. The first successful use of beclomethasone dipropionate (BDP) as a pressurized aerosol with no apparent evidence of systemic toxicity was published in 1972 [5]. In the years since, corticosteroid molecules have been refined to create more potent agents with lower bioavailability and enhanced safety profiles. Currently, 8 INS compounds are approved for the management of allergic rhinitis (AR) in the United States: BDP, budesonide, ciclesonide, flunisolide, fluticasone furoate, fluticasone propionate (FP), mometasone furoate, and triamcinolone acetonide (Table 1) [6].

**Table 1:** Available Intranasal Corticosteroids

Generic (Proprietary) Name	Recommended Dosage
Beclomethasone dipropionate	*Adults and children *> *12 years of age: *1 or 2 sprays (42 to 84 *μ*g) per nostril BID (total dose 168 to 336 *μ*g/d)
(Beconase AQ)	*Children 6-12 years: *1 spray (42 *μ*g) per nostril BID for total of 168 *μ*g/d up to 2 sprays per nostril BID for total of 336 *μ*g/d
Budesonide (Rhinocort Aqua)*	*Adults and children *> *6 years of age: *1 spray (32 *μ*g/spray) per nostril QD up to a maximum of 256 *μ*g/d (> 12 years of age) or 128 *μ*g/d (6 to < 12 years of age)
Ciclesonide (Omnaris)	*Adults and children *> *12 years of age: *2 sprays (50 *μ*g/spray) per nostril QD
Flunisolide (Nasarel)	*Adults: *2 sprays (58 *μ*g) per nostril BID, not to exceed 8 sprays per nostril per day (464 *μ*g)
	*Children 6-14 years of age: *1 spray (29 *μ*g) per nostril TID or 2 sprays (58 *μ*g) per nostril BID, not to exceed 4 sprays per nostril per day (232 *μ*g)
Fluticasone furoate (Veramyst)	*Adults and children *> *12 years of age: *2 sprays (55 *μ*g) per nostril QD
	*Children 2-11 years of age: *1 spray (27.5 *μ*g) per nostril QD up to 2 sprays (55 *μ*g) per nostril QD
Fluticasone propionate (Flonase)	*Adults: *2 sprays (100 *μ*g) per nostril QD or 1 spray (50 *μ*g) BID
	*Adolescents and children > 4 years of age: *1 spray (50 *μ*g) per nostril per day up to, but not in excess of, 2 sprays (100 *μ*g) per nostril per day
Mometasone furoate (Nasonex)	*Adults and children *> *12 years of age: *2 sprays (100 *μ*g) per nostril QD
	*Children 2-11 years of age: *1 spray (50 *μ*g) per nostril QD
Triamcinolone acetonide	*Adults and children *> *12 years of age: *2 sprays (110 *μ*g) per nostril QD
(Nasacort AQ)	*Children 6-12 years of age: *1 spray (55 *μ*g) per nostril or 110 *μ*g QD, up to 2 sprays (110 *μ*g each) per nostril or 220 *μ*g QD

*Available in 2 strengths: 32 and 64 *μ*g per spray.

**Figure 1:**
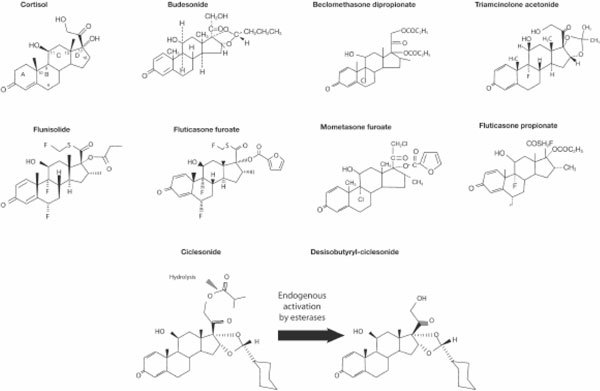
**Chemical structures of intranasal steroids **[6].

Corticosteroid molecules are derived from the parent molecule, cortisol [7]. The carbon framework of each corticosteroid is made up of three 6-carbon rings as shown in Figure 1 (rings A, B, and C) and one 5-carbon ring (ring D) [6]. All anti-inflammatory corticosteroids have features in common with cortisol and with each other: a ketone oxygen at position 3; an unsaturated bond between carbons 4 and 5; a hydroxyl group at position 11; and a ketone oxygen group on carbon 20. The variations occurring off ring D at positions 16, 17, and 21 are the greatest differentiating factors between the individual molecules. Structure-activity relationship studies of this region led to the identification of chemical groups that enhance topical activity and reduce systemic adverse events [4]. For example, the furoate group of mometasone furoate was found to enhance molecular affinity for the glucocorticoid receptor binding site.

Other modifications have improved the activity of corticosteroid compounds. The 21-chloro 17 (2' furoate) group on the mometasone furoate structure improves anti-inflammatory activity, whereas the chloride at position 21 provides the additional benefit of inferring resistance to degradation by esterases [4]. Halogen substitutions at positions 6 and 9 are thought to increase potency, as are side-chain substitutions at position 17 [8].

The pharmacotherapeutic objective of INS is to down-regulate the recruitment and influx of inflammatory cells and inhibit the secretion of pro-inflammatory mediators during the inflammatory response [9]. This process is evidenced in AR by reduced levels of histamine, leukotrienes, and mast cells recovered in the nasal fluid and mucosa of patients treated with INSs [9-12].

## Mechanisms of action of intranasal corticosteroids

### T-lymphocytes

Corticosteroid therapy provides a modest reduction in the number of lymphocytes because of induction of programmed cell death or apoptosis [13]. Topical administration has been shown to inhibit T-lymphocyte activation, prevent increases in interlukin-4 (IL-4), IL-5, and local IgE and inhibit eosinophil recruitment and activation,[14,15] IL-2 production,[16] and IL-2 generation [17].

### Eosinophils

Corticosteroids have well recognized effects on the eosinophil component of the inflammatory process by direct induction of eosinophil apoptosis [18] and inhibition of eosinophil recruitment and migration into the nasal airways. Cytokine production in the airways may trigger this cellular infiltration, and inhibition of cytokine production is, therefore, one of the most important effects of corticosteroid therapy [19]. Corticosteroids are effective and potent inhibitors of cytokines such as tumor necrosis factor alpha and IL-1, which induce secretion of the nonspecific endothelial activators [20]. They also inhibit the release of IL-4 and IL-13, thereby preventing expression of specific endothelial cell adhesion molecules, which can bind basophils, eosinophils, monocytes, and lymphocytes [14]. Corticosteroid treatment has been shown to inhibit the expression of the chemokine RANTES in airway epithelial cells [21]. Furthermore, corticosteroids inhibit the production of IL-3, IL-5, and granulocyte-macrophage colony-stimulating factor (GM-CSF)[22] and reduce eosinophil survival time [23].

Thus, corticosteroids have multiple effects on eosinophils; they reduce the number of eosinophils in the circulation, prevent recruitment of eosinophils to local tissue sites,[24] reduce survival of eosinophils, and prevent production of cytokines that are responsible for, or involved in, all of these processes. Indeed, corticosteroids inhibit the production of many cytokines that may explain much of their anti-inflammatory activity.

### Mast Cells and Basophils

Topical corticosteroids can reduce inflammation by affecting the infiltration of mast cells and basophils. Treatment can decrease the number of mast cells in the nasal mucosa [25,26]. Corticosteroid treatment has also been shown to markedly reduce the number of basophils in nasal secretions [27]. An associated reduction in the release of mast cell mediators has been demonstrated after corticosteroid treatment and interference with arachidonic acid metabolism and a subsequent decrease in mediator production [28,29].

### Neutrophils

Corticosteroids are able to inhibit the accumulation of neutrophils,[30] possibly because of preventing neutrophil adherence to vascular endothelium by suppressing the release of endothelial-activating cytokines, for example, IL-1, IL-4, and tumor necrosis factor alpha, and preventing the release of factors that influence migration through the endothelial barrier, for example, IL-8, tumor necrosis factor alpha, platelet activating factor (PAF), and LTB4 [31].

### Monocytes and Macrophages

Corticosteroids reduce the number of tissue macrophages and inhibit their release of IL-1, interferon gamma, tumor necrosis factor alpha and GM-CSF (Table 2) [19,32].

**Table 2:** Effects of Corticosteroids on Inflammatory Cells

Cells Affected	Corticosteroid Effect
*T-lymphocytes*	Reduction of circulating cell number, apoptosis
	Inhibition of:
	*T-lymphocyte *activation
	IL-2 production
	IL-2 receptor generation
	IL-4 production
	Antigen-driven proliferation
Eosinophils	Reduction of circulating cell number, apoptosis
	Reduction of epithelial and mucosal cell counts
	Reduction of cell influx in late-phase response
	Inhibition of IL-4 and IL-5-mediated cell survival
Mast cells/basophils	Reduction of circulating cell counts
	Reduction of cell influx in late-phase response
	Reduction of mast cell-derived mediators after challenge
	Reduction of histamine content and release
Neutrophils	Reduction of cell influx after challenge
Macrophages/monocytes	Reduction of circulating cell counts
	Inhibition of release of:
	IL-1
	Interferon-gamma
	TNF-alpha
	GM-CSF

### Epithelial Cells

Human epithelial cells metabolize arachidonic acid. Arachidonic acid metabolites, such as leukotrienes, prostaglandins, thromboxane, and PAF, have several activities, including regulation of vascular tone and vascular permeability, stimulation of mucus secretion, chemotaxis, and regulation of cell proliferation. The cytokines produced by epithelial cells can also mediate recruitment, activation, and survival of inflammatory cells in the airway [33].

Corticosteroids interfere with the inflammatory response by blocking the production of arachidonic acid metabolites in many cells. This is accomplished by inducing the production of the anti-inflammatory protein, lipocortin, which inhibits phospholipase A2, a central enzyme in the arachidonic acid cascade, thereby preventing the generation of cyclo-oxygenase and lipoxygenase products [33].

### Blood Vessels

Topical corticosteroids reduce blood flow and inhibit vascular permeability. The mechanisms for these actions include a reduction in the cyclo-oxygenase metabolites that maintain vascular beds, inhibition of phospholipase A2, and the subsequent formation of leukotrienes and PAF,[34] inhibition of the release of endothelial-derived relaxing factor, production of vasocortin (which reduces permeability), and enhancement of vasospasm by alpha adrenergic stimulation [35,36].

### Nerve Cells

Steroids have been reported to up-regulate neutral endopeptidase that degrades neuropeptides [37] and inhibits neurogenic plasma extravasation [38].

## The glucocorticoid receptor

Corticosteroid activity is mediated by intracellular activation of the glucocorticoid receptor (GR) [39,40]. In its inactive state, the GR exists as a cytosolic protein bound to 2 heat shock protein 90 chaperonin molecules. Binding to the corticosteroid ligand results in a conformational change that allows dissociation of the GR from the protein complex, and a quick translocation into the cell nucleus. The ligand-bound GR can modulate gene expression in the nucleus by binding to glucocorticoid response elements in promoter regions of responsive genes. The GR binds to the glucocorticoid response elements as a homodimer and acts as a transcription factor. It has also become evident that the GR can regulate gene expression unfacilitated by glucocorticoid response elements through direct interaction with transcription factors such as nuclear factor NF-kappa B and activating protein [39,41,42]. The inhibition of these 2 factors leads to down-regulation of the production of cytokines and other inflammatory molecules and is thought to be among the primary mechanisms for the anti-inflammatory effects of corticosteroids [39,40].

## Pharmacodynamic properties of Intranasal Corticosteroids

**Figure 2:**
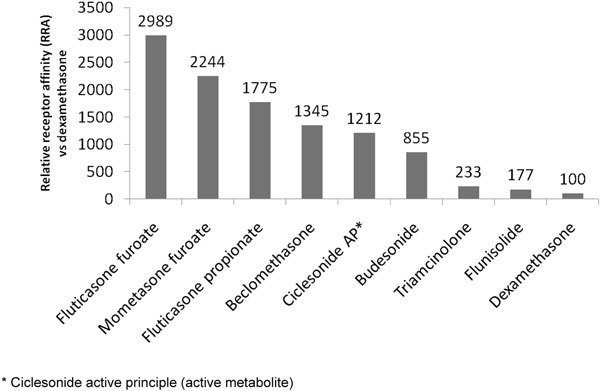
**Relative glucocorticoid receptor affinity of intranasal corticosteroids **[6].

Glucocorticoid potency can be measured in various ways, but is thought to be closely related to GR binding affinity [6]. Figure 2 illustrates the relative GR binding affinities for most intranasal compounds. Relative receptor affinity studies are highly dependent on assay methodology and prone to error [43]. Although specific values vary between studies, most have shown a similar order of potency. An earlier study of INSs that used competition assay methodology reported a similar rank order of relative binding potency: mometasone furoate > FP > budesonide > triamcinolone acetonide > dexamethasone [40].

Corticosteroid potency also has been evaluated using the McKenzie assay, which compares the relative cutaneous vasoconstrictor and skin blanching responses of individual compounds [44]. Using this methodology, the rank order of potency was: FP > mometasone furoate > budesonide > flunisolide > triamcinolone acetonide (Table 3) [19,45]. This vasoconstriction phenomenon, which occurs in all subjects, has been suggested as part of the explanation of the glucocorticoid effect on the nasal mucosa. In a randomized double-blind cross-over study, using the 133Xe wash-out method as a measure of nasal mucosal blood flow, the effect of topically administered budesonide was compared with placebo. No difference between the groups was found to occur. It seems likely that a more complex activity than vasoconstriction is responsible for the clinical effect of INS [46].

**Table 3:** Potency of Topical Corticosteroids Based on Receptor Binding Affinity and the Skin-Blanching Test

Corticosteroid	Recptor Binding Affinity*	Skin Blanching Potency*
Flunisolide	1.8	330
Triamcinolone acetonide	3.6	330
BDP	0.4	600
Budesonide	9.4	980
FP	18.0	1200

Results of studies using another marker of corticosteroid potency, transactivation potency, correlate with receptor binding affinity results. One study found mometasone furoate the most potent GR ligand, requiring the lowest concentration to affect 50% of the maximum level of transcription activation of a glucocorticoid response element reporter gene in cells [40]. Overall rank of potency using this methodology was: mometasone furoate > FP > triamcinolone acetonide > budesonide > dexamethasone.

There is no evidence of a linear association between glucocorticoid potency and clinical response, nor is there a known "plateau" beyond which greater potency does not add additional benefit. Likewise, it is not evident that the compound with the highest receptor affinity will have superior clinical efficacy. Although increased potency at intranasal sites would seem desirable, the possibility of greater potency at other sites could theoretically increase the risk of systemic adverse effects, because GRs are similar throughout the body.

## Clinical Implications

### Onset of Action

It was traditionally accepted that INSs should be used for days or weeks to achieve significant benefits, and because these agents were used primarily to control chronic symptoms, few studies focused on onset of action [47]. Symptom improvement has been noted within 1 to 2 days after administration of most newer agents [47]. It is not uncommon for patients to use intranasal medications intermittently on an "as-needed" basis,[47] and such use may be justified given the potential for a more rapid onset of action than was previously ascribed to these medications.

### Clinical Efficacy

The design of topically active INS formulations has provided a much better therapeutic ratio than oral corticosteroids [36]. The pharmacodynamic and pharmacokinetic properties of these agents play an important role in facilitating local anti-inflammatory activity with a low rate of side effects. However, it remains to be seen whether the often subtle pharmacodynamic and pharmacokinetic differences between the compounds distinguish them in a clinical setting.

Based on currently available data, there is no clear evidence that any INS is superior to any other for AR symptom relief [47,48] despite the pharmacologic differences between many of them. It is possible that the degree of anti-inflammatory activity required for AR symptom relief is low enough that relief is easily achieved by agents of varying potencies, or that GR saturation or near saturation occurs with all of the preparations [47,49]. These considerations have not been well studied in the treatment of VMR.

## Intranasal Corticosteroid Therapy for Vasomotor Rhinitis

INSs are one of the classes of agents that have received US Federal Drug Administration approval for use in nonallergic rhinitis. Specific formulations that are approved are BDP aqueous, budesonide aerosol, and FP aqueous. It is assumed that the anti-inflammatory activities of the INSs account for their beneficial effect in VMR [50].

### BDP

BDP was first reported to be effective for VMR in 1976 [50]. In Sweden 39 male and female patients aged 19-66 years (average, 39 years) with perennial nasal obstruction, drip, itching, and sneezing were studied in a randomized, double-blind, placebo-controlled, 4-week treatment period crossover designed trial. Intracutaneous specific aeroallergen testing for these subjects was negative, but serum total IgE ranged from < 100 ng/ml (N = 21), 101-200 ng/ml (N = 8), 200-400 ng/ml (N = 6), 401-900 ng/ml (N = 3), to 1200 ng/ml (N = 1). Nasal secretion eosinophilic cells were estimated to be < 10%. The dosage was one aerosol-delivered puff into each nostril 3 times a day (300 *μ*g of BDP per day during the active period) [51].

There was no difference in the response of the groups on the first day of treatment. However, a significant reduction in the total symptom score was observed during the second week of the BDP treatment period. This effect remained throughout the rest of the period. For the individual nasal symptoms, a significantly lower score for the BDP period was registered for sneezing during the first week, for nasal drainage during the second week, for nasal itching during the third week, and for nasal blocking only during the fourth week of treatment (Table 4) [51].

**Table 4:** Results During Treatment Periods With Beclomethasone and Placebo

Sneezing	Day 1	n.s.
	Week 1	*P *< 0.05
	Week 2	*P *< 0.01
	Week 3	*P *< 0.01
	Week 4	*P *< 0.01
Nasal catarrh	Day 1	n.s.
	Week 1	n.s.
	Week 2	*P *< 0.01
	Week 3	*P *< 0.01
	Week 4	*P *< 0.01
Itching	Day 1	n.s.
	Week 1	n.s.
	Week 2	n.s.
	Week 3	*P *< 0.05
	Week 4	*P *< 0.05
Blocking	Day 1	n.s.
	Week 1	n.s.
	Week 2	n.s.
	Week 3	n.s.
	Week 4	*P *< 0.01
Total score	Day 1	n.s.
	Week 1	n.s.
	Week 2	*P *< 0.01
	Week 3	*P *< 0.01
	Week 4	*P *< 0.01

*P*-values favor treatment with beclomethasone.

n.s., not significant.

On questioning, after the BDP period, 4 patients considered themselves free of trouble, 25 had improved, 10 were unchanged, and no one had worsened; 74% considered themselves free of symptoms or greatly improved. After the placebo period, no one was free of trouble, 12 had improved, 20 were unchanged, and 7 patients had worsened; 31% considered themselves free of symptoms or greatly improved. Of the 39 who completed the study, 25 preferred the BDP period, 5 preferred the placebo period, and 9 found no difference between the periods. The authors concluded that the results were encouraging because approximately 75% of the patients considered themselves free of trouble or improved when treated with the intranasal corticosteroid, BDP [51].

A second Swedish study on the effects of BDP aerosol for VMR was reported from a dose-ranging, crossover, double-blind, placebo-controlled trial [52]. Subjects, 18-61 years (average, 36 years), with persistent nasal symptoms were studied by skin prick testing and provocation testing. No relevant allergic sensitivities were found in the 21 subjects. Each group took a dose (given in a twice a day regimen) in a randomized fashion of placebo and 200, 400, and 800 *μ*g of the active drug during four 2-week periods. To avoid or reduce the effects of BDP lingering on from previous periods of treatment, the statistical evaluation of the symptoms was limited to the last week of each period [52].

**Figure 3:**
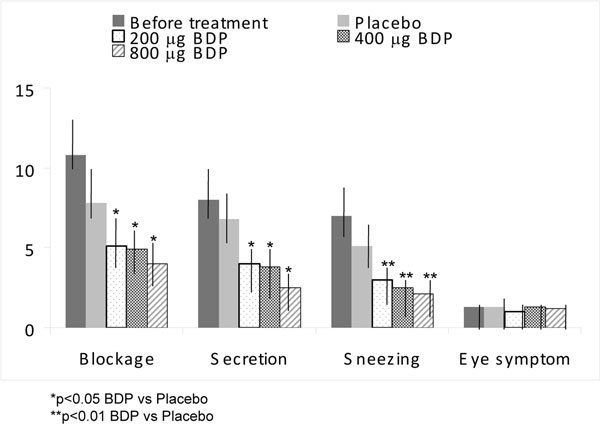
**Efficacy of BDP aerosol for VMR**. Symptom scores in each second week of every 2-week period of crossover trial and the pretrial week (mean ± SEM) [52].

Although no differences in efficacy were determined between the 3 BDP doses, all doses of BDP aerosol were significantly more effective than placebo in reducing nasal blockage, nasal secretions, and sneezing (*P *< 0.05, Figure 3) [52]. During the week before treatment with placebo and BDP, the patients used altogether 80 antihistamine tablets. During the week of treatment with placebo, the number dropped to 20 tablets; and during the weeks with 200, 400, and 800 *μ*g BDP, 13, 8, and 9 tablets were taken, respectively. Evaluations of nasal secretions revealed that 18 of 21 patients had more than 10% eosinophils in the week before treatment. During the periods of treatment, the percentage of eosinophils was reduced to 10% with placebo, and to 8, 5, and 8% during treatment with 200, 400, and 800 *μ*g BDP, respectively. The authors recommended that the lowest effective dose of BDP be prescribed [52]. Similar results were reported for a once versus twice daily BDP dose study for the treatment of VMR [53].

### Budesonide

Budesonide aerosol was investigated to determine its effect on the symptoms of patients with perennial nonallergic rhinitis [54]. Participants in the study were 22 patients aged 20-68 years (average, 42 years). All the patients had negative prick test reactions to standard allergens. Analysis of serum IgE levels showed 9 patients < 20 kU/l and 20-100 kU/l in 13 patients. The trial had a double-blind, cross-over design randomizing the twice a day dosing of placebo 50, 200, and 800 *μ*g of budesonide with each preparation taken for 2 weeks [54].

**Figure 4:**
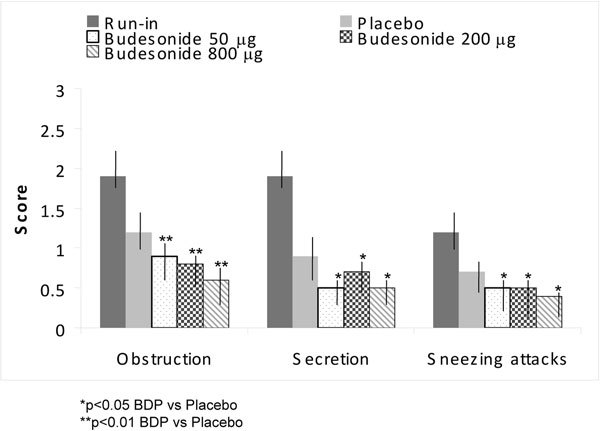
**Efficacy of budesonide for VMR**. Symptom scores (mean ± SEM) in budesonide versus placebo for each 2-week crossover period [54].

Budesonide was significantly more effective than placebo with regard to the symptoms of nasal obstruction, nasal drainage, and sneezing (*P *< 0.05). No significant differences were established between the budesonide dosages (Figure 4) [54].

Objective parameters were also evaluated. Nasal airway resistance was determined by means of anterior rhinomanometry. There were no differences in respect to nasal patency between the sitting and recumbent positions, between the placebo and budesonide groups and between the dosages within budesonide (Table 5) [54]. Nasal eosinophilia (> 10% of leukocytes) was present among 10 of 22 patients in the run-in period. Eosinophilia in nasal smears was reduced by budesonide (*P *< 0.01). Nasal secretion was induced by the intranasal delivery of 0.2 ml metacholine bromide solution in each nostril using a De Vilbiss #15 spray. A total of 0.4 ml containing 12 mg metacholine was given. Secretions were then collected for 15 minutes through a funnel held under the subject's nose. The metacholine induced nasal secretion was significantly lowered by all the treatments as compared with the amount induced during the run-in period. Budesonide, in dosages of 200 and 800 *μ*g daily significantly reduced the metacholine-induced secretion as compared with the placebo treatment (Table 6) [54].

**Table 5:** Rhinomanometry: Nasal Resistance Parameter V_2 _(Degrees)

	Mean ± SEM	
	
	Sitting	Recumbency
Run-in	49 ± 4	49 ± 5
Placebo	45 ± 4	45 ± 4
Budesonide 50 *μ*g	42 ± 4	41 ± 4
Budesonide 200 *μ*g	40 ± 3	39 ± 3
Budesonide 800 *μ*g	41 ± 4	40 ± 3
Significances from ANOVA		
Run-in vs placebo and budesonide	*P *< 0.05	*P *< 0.05
Placebo vs budesonide	n.s.	n.s.
Dosages within budesonide	n.s.	n.s.

n.s., not significant.

**Table 6:** Methacholine-Induced Nasal Secretion (ml/15 Minutes)

	Mean	Median	Median Decrease Relative to Run-in	Median Decrease Relative to Placebo
Run-in	0.89	0.59		
Placebo	0.56	0.33	0.18 (*P *< 0.05)	
Budesonide 50 *μ*g	0.38	0.31	0.42 (*P *< 0.01)	0.12 n.s.
Budesonide 200 *μ*g	0.31	0.35	0.42 (*P *< 0.001)	0.09 (*P *< 0.05)
Budesonide 800 *μ*g	0.29	0.20	0.37 (*P *< 0.01)	0.06 (*P *< 0.05)

n.s., not significant.

Budesonide aerosol, 2 puffs twice a day, was also studied in 12 subjects with an age range of 22-53 years (mean age, 37 years) in a long-term open uncontrolled trial of patients with vasomotor rhinitis. All subjects were skin test negative [55]. Clinical evaluations were performed after 1, 2, 4, 6, 9, and 12 months. All patients completed the 1-year trial period. A nasal biopsy was obtained before beginning medication and after 1 year of treatment.

**Figure 5:**
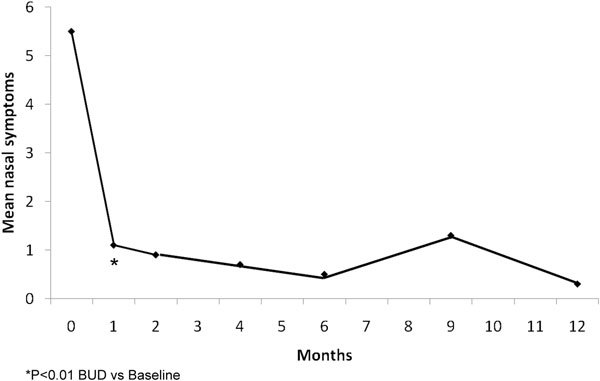
**Efficacy of budesonide aerosol 200 *μ*g twice daily, on symptoms of VMR in a long-term (1 year) open uncontrolled trial**. Mean nasal symptom scores by month [55].

Symptom scores were substantially decreased by the 1 month appointment and this improvement persisted for the whole year (Figure 5) [55]. No increase in the daily dose was noted; in fact, 3 patients, on their own, halved their daily intranasal steroid dose from 400 to 200 *μ*g without any recurrence of symptoms. The decrease of symptoms compared with the entry values was statistically significant (*P *< 0.01). The differences between the baseline and the end of treatment biopsies were in most cases nonexistent or very small (Table 7) [55]. In only 3 cases did any of the 8 variables change by more than one degree. The authors conclude that VMR, a "pathologic puzzle," can be successfully treated with INS [55].

**Table 7:** Microscopic Investigation Before (I) and After (II) Treatment

	Patients											
	
Variables	1	2	3	4	5	6	7	8	9	10	11	12
Squamous epithelium												
I	-	0	-	++	++	0	0	0	+++	0	0	-
II	+	0	0	++	+	0	0	++	++	0	0	0
Goblet cells												
I	-	++	-	+	+	+	+++	0	0	0	++	-
II	0	++	++	+	+	+	+++	0	++	++	+	++
Inflammatory cells in epithelium												
I	-	0	-	+	+	++	+	+	+	+	+	-
II	0	0	0	+	0	++	0	+	+	+	+	+
Thickening of the basal lamina												
I	++	++	+	+	0	++	++	+	+	+	++	+
II	++	++	++	+	+	+	++	+	+	++	+	+
Eosinophils												
I	0	+	0	+	+	+	+	0	++	+	0	0
II	0	0	0	0	0	+	0	0	++	+	+	+
Neutrophils												
I	0	0	+	+	+	++	+	0	+	+	0	0
II	0	0	+	0	++	++	0	0	+	+	+	0
Plasma cells												
I	0	+	0	+	++	+	0	0	++	+	0	0
II	+	++	0	+	+	+	0	0	++	+	+	0
Lymphocytes												
I	+	+	+	+	++	+	+	+	+	+	+	+
II	++	+	+	+	+	+	+	+	+	+	+	++

**Figure 6:**
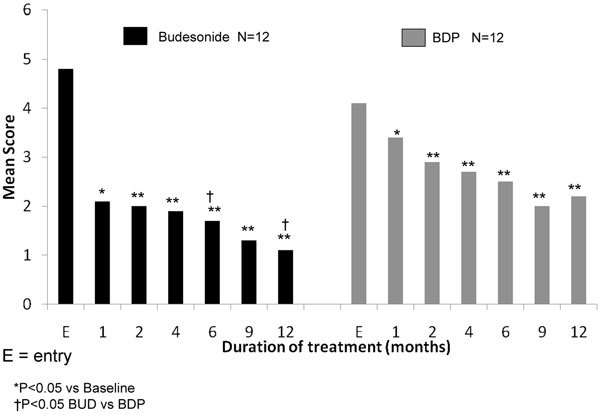
**Comparison of the efficacy of budesonide and BDP aerosols in doses of 400 *μ*g/d in a long-term study of 24 patients with perennial nonallergic rhinitis**. Nasal symptom scores more than 12 months (mean ± SEM) [56].

The efficacy of budesonide and BDP aerosols in doses of 400 *μ*g/d were compared in a long-term study of 24 patients with perennial nonallergic rhinitis [56]. The total nasal symptom score showed a statistically significant decrease from baseline at all visits in both groups, but at 6 and 12 months the reduction was significantly greater (*P *< 0.05) in the budesonide treatment group (Figure 6) [56].

The Agency for Healthcare Research and Quality reviewed one double-blind, placebo-controlled study with 2 doses of budesonide for nonallergic rhinitis. They found it showed significant improvement in nasal obstruction [57].

### FP

FP nasal spray has been shown to have a beneficial effect on inflammatory cells and cytokine profile. Application of this product resulted in decreases in the number of CD3+ cells, the amount of major basic protein, and the number of tryptase-positive cells in subjects with nonallergic rhinitis [58]. In addition, the use of this agent effectively lowered messenger RNA expression for IL-4 and IL-5 [58].

**Figure 7:**
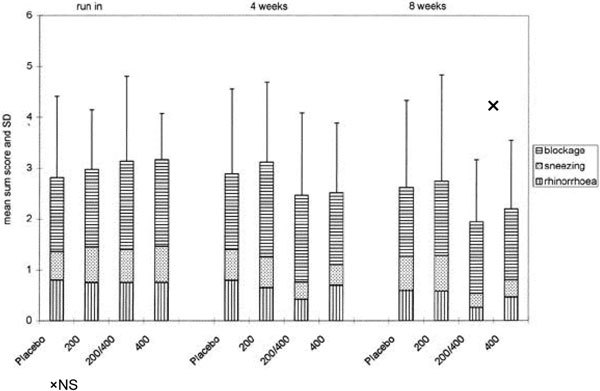
**Comparison of different treatment regimens of FP aqueous nasal spray (FPANS) 200 *μ*g (once daily for 8 weeks, twice daily for 8 weeks, once daily for 4 weeks followed by twice daily for 4 weeks) in patients with nonallergic noninfectious rhinitis**. Sums of the scores of blockage, sneezing, and rhinorrhea (mean ± SD) [3].

In a study of 65 patients with nonallergic noninfectious rhinitis, comparing 8 weeks of placebo with FP aqueous nasal spray 200 *μ*g once daily, FP 200 *μ*g twice daily, and FP once daily for 4 weeks followed by FP twice daily for 4 weeks, no significant changes were seen for the mean sums of the scores of blockage, sneezing, and rhinorrhea (Figure 7) [3]. No significant difference between the 4 groups was found in the before treatment biopsies. A significant dose dependent decrease in the immunocompetent cells was found after treatment. In the epithelium, a marked decrease in the number of Langerhans cells and T cells was seen. In the lamina propria, at the end of treatment, the mast cells and eosinophils (rarely found) were reduced if present at baseline. The authors concluded that FP did not provide symptom improvement and the significant dose-dependent steroid effect on immunocompetent cells does not seem to be a clinically relevant benefit [3].

A placebo controlled trial with FP, studied 35 patients with hypertrophic inferior turbinates and a diagnosis of VMR based on nasal symptoms, negative skin prick allergy tests, and negative nasal cytologic examination for eosinophils [59]. The dosage was administered as 1 spray/nostril twice a day, giving a daily dose of 200 *μ*g of FP.

**Figure 8:**
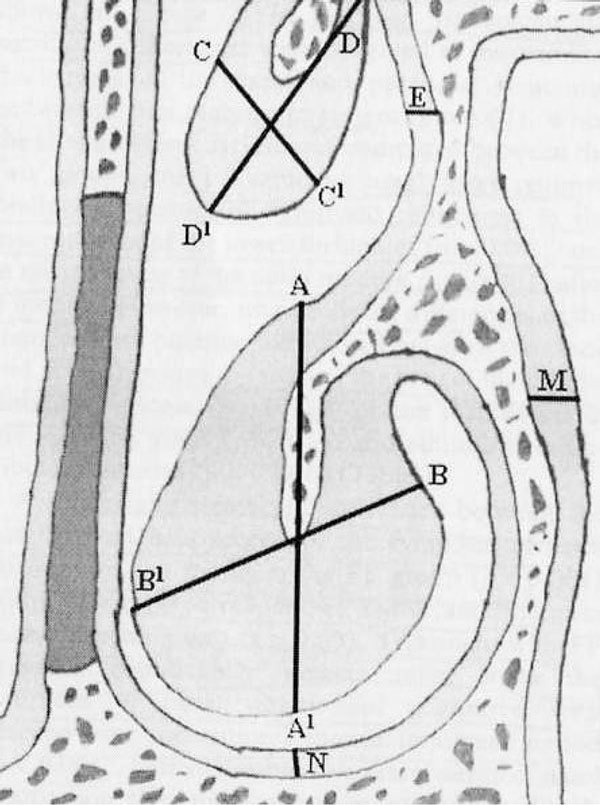
**Method for measuring area of turbinate mucosa and thickness of nasal, maxillary sinus, and ethmoidal infindibulum mucosa **[59].

Treatment with FP provided significantly greater relief from the symptom of nasal obstruction compared with placebo over the entire 3 month treatment period (*P *< 0.001). The FP nasal spray regimen produced statistically significant reductions in the mucosal area of the lower turbinates (*P *= 0.002) and in the thickness of the nasal mucosa (*P *< 0.001) after 3 months compared with placebo treatment as assessed by means of computed tomographic (CT) scanning (Figure 8) [59]. However, no significant group differences were seen for the middle turbinate mucosa, or the thickness of the maxillary sinus or the ethmoid infindibulum mucosae.

**Figure 9:**
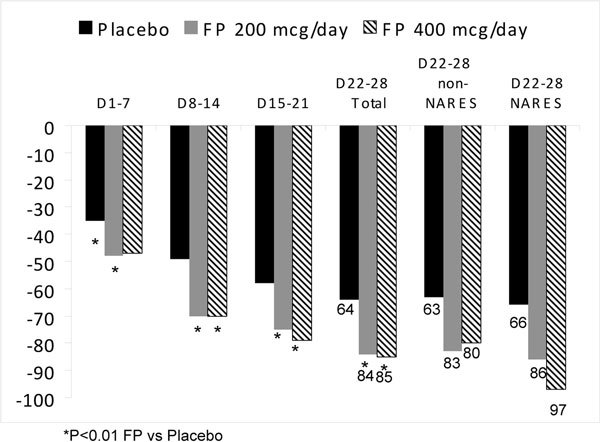
**Placebo-controlled comparison of FP nasal spray given as a AM/PM divided dose of 200 and 400 *μ*g**. Patient-rated mean total nasal symptom scores (TNSS) in total, non-NARES, and NARES populations [60].

FP nasal spray in an AM PM divided dose of 200 and 400 *μ*g was compared with placebo in a large trial of 983 patients with perennial nonallergic rhinitis as documented by history and negative skin tests to all allergens relevant to the region [60]. Cytologic examination showed that ~31% of the subjects had prominent nasal eosinophilia (NARES syndrome) and ~69% did not have nasal eosinophilia (non-NARES, or vasomotor). Both FP study groups demonstrated significantly greater reduction in their total nasal symptom score (TNSS) compared with placebo over the 28 day treatment period (*P *< 0.01). The mean changes for all patients treated with FP 200 and FP 400 was -84 and -85, respectively (maximum visual analogue score of 300). For placebo, the mean change in TNSS was -64. There were no statistical differences between the 2 FP treatment groups at any time point (Figure 9) [60]. In the NARES subgroup, mean changes in TNSS for the FP 200 *μ*g and placebo groups were similar to changes seen in the total population (-86 and -66 respectively); mean change in the TNSS for the FP 400 *μ*g group was somewhat greater (-97) than change seen in the total population. In the non NARES subgroup, mean changes in TNSS for each treatment group were similar to changes seen in the total population (-83 and -80 in the FP 200 *μ*g and FP 400 *μ*g groups, respectively, as opposed to -63 in the placebo group) (Figure 9) [60]. The authors concluded that FP is an effective treatment for perennial nonallergic rhinitis with or without eosinophilia.

### Mometasone Furoate

The benefit of INS in perennial nonallergic rhinitis has been inconsistent. A study of mometasone furoate nasal spray (MFNS) 200 *μ*g once daily showed improvement rates to be numerically better (56% vs. 49%) but not statistically significantly more effective than placebo (*P *= 0.25) in reducing overall rhinitis symptoms in a cohort of 329 subjects with nonallergic rhinitis during a 6 week treatment period [61]. However, during the follow-up period with no treatment the relapse rates (increase in any score > 1) in the subset of improved patients were significantly higher in the MFNS groups for both subject's [*P *= 0.11 (intention-to-treat), *P *= 0.04 (per protocol)] and investigator's [*P *= 0.03 (intention-to-treat), *P *= 0.01 (per protocol)] overall evaluations [61].

BDP (400 *μ*g/d) and the intranasal anticholinergic agent, ipratropium bromide (160 *μ*g/d) aerosol preparations were compared in a cross-over trial in 24 patients (ages 20-77 years) with nonallergic rhinitis (negative skin prick tests for allergic reactivity) with excessive nasal secretions [62]. Significant differences between the treatments were not found in the number of paper tissues used nor in the scorings of nasal secretions, sneezes, and amount of nasal blockage (Table 8) [62]. Nasal secretions tended to be more reduced by ipratropium and nasal blockage and sneezing reduced more by beclomethasone. Eight patients had eosinophilia in the nasal smear. Three of them preferred ipratropium and 5 beclomethasone. In the 16 patients without nasal eosinophilia, 11 preferred ipratropium and 4 beclomethasone. Of the 24 patients, 14 preferred ipratropium, 9 preferred BDP, and 1 subject had no preference (not statistically significant). The authors concluded that it was not possible to characterize patients who would necessarily benefit with either ipratropium or BDP [62].

**Table 8:** Nasal Symptom Scores and Number of Additional Doses for the Period of 2 Weeks

	Ipratropium Mean ± SEM	Beclomethasone Mean ± SEM	Max. Score (2 Weeks)
Nasal secretion	18.0 ± 2.5	19.8 ± 3.3	(56)
Sneezings	12.8 ± 1.8	11.4 ± 1.9	(42)
Nasal blockage	8.0 ± 2.0	6.1 ± 1.8	(42)
Number of paper tissues	110.6 ± 15.8	111.8 ± 16.0	
Additional doses	19.0 ± 2.8	19.0 ± 3.2 (placebo)	

Administration of INS therapy in combination with other agents has been suggested for treatment of nonallergic rhinitis. Suggestions include a topical steroid plus a topical antihistamine [63] and topical steroid plus topical ipratropium bromide [64].

## Conclusions

INSs have numerous pharmacologic properties. These include the ability to reduce inflammatory cells, cytokines, and mediators, and their consequent effects on blood vessel, glands, and nerves. As the pathophysiology of VMR is not fully understood, there is the possibility that INSs could offer therapeutic benefits. Studies to date, whereas not always similarly designed and not always consistent in results, generally suggest that treatment of this condition with INSs has value.

Further well planned clinical trials of these agents for VMR are warranted. Endpoints that have been used in past studies and should be considered in the future include:

• Quantifying symptom scores--total and individual components

• Quantifying the perception of degree of overall improvement

• Quantifying use of rescue medicine

• Assessing relapse rates after treatment cessation

• Assessing impact on quality of life

• Counting number of required paper tissues

• Measuring metacholine-induced nasal secretions

• Measuring nasal resistance/airway patency

• Distinguishing responses in patients with low, normal, and high serum IgE levels

• Distinguishing responses in patients with non-NARES and NARES cytologic patterns

• Evaluating biopsy patterns pre- and posttreatment

• Assessing CT scans of inferior turbinates and nasal mucosae

• Assessing differences between agents in class

• Assessing any significant dose effect

• Correlating subjective and objective findings

## End Note

Received grant/research support from Alcon, Amgen, Apotex, AstraZeneca, Boehringer Ingelheim, Capnia, Genentech, GlaxoSmithKline, MAP Pharmaceuticals, Meda, Merck, Novartis, Pharmaxis, SanofiAventis, Schering-Plough, Sepracor, Skye Pharma, Teva, Vocel, and Wyeth. He is a consultant, or on an advisory board or the speakers bureau for Abbott, Alcon, Amgen, AstraZeneca, Capnia, Dey Labs, Genentech, GlaxoSmithKline, Meda, Merck, Novartis, Pharmaxis, SanofiAventis, Schering-Plough, Sepracor, Vocel, and Wyeth.

Presented at a roundtable conference held in December 2008 in Washington, DC. The meeting was sponsored by the TREAT Foundation (Washington, DC) and supported through an unrestricted educational grant from Meda Pharmaceuticals. The funding company did not have any input into the development of the meeting or the series, and the company was not represented at the roundtable meeting.
